# ER+/PR− phenotype exhibits more aggressive biological features and worse outcome compared with ER+/PR+ phenotype in HER2-negative inflammatory breast cancer

**DOI:** 10.1038/s41598-023-50755-4

**Published:** 2024-01-02

**Authors:** Yunbo Luo, Qingyun Li, Jiang Fang, Chaoying Pan, Lingxing Zhang, Xia Xu, Shuangqiang Qian, Xiaobo Zhao, Lingmi Hou

**Affiliations:** 1 Department of Thyroid and Breast Surgery, Affiliated Hospital of North Sichuan Medical College, Nanchong, Sichuan China; 2 Department of Thyroid and Breast Surgery, Guigang City People’s Hospital, The Eighth Affiliated Hospital of Guangxi Medical University, Guigang, Guangxi China; 3 Department of Academician (Expert) Workstation, Biological Targeting Laboratory of Breast Cancer, Breast and Thyroid Surgery, Affiliated Hospital of North Sichuan Medical College, Nanchong, China

**Keywords:** Breast cancer, Tumour heterogeneity

## Abstract

The loss of progesterone receptor (PR) often predicts worse biological behavior and prognosis in estrogen receptor-positive (ER +) breast cancer. However, the impact of PR status on inflammatory breast cancer (IBC) has not been studied. Therefore, the purpose of our study was to investigate the influence of PR on IBC. Patients with ER+ and HER2-negative IBC were selected from the Surveillance, Epidemiology and End Results database. Pearson’s χ^2^ test was used to compare the clinicopathological characteristics between patients with estrogen receptor-positive/progesterone receptor-positive (ER+/PR +) and patients with estrogen receptor-positive/progesterone receptor-negative (ER+/PR−). Univariate and multivariate analyses were performed to investigate the effects of PR status on the breast cancer-specific survival (BCSS) and overall survival (OS) in IBC. Overall, 1553 patients including 1157 (74.5%) patients with ER+/PR+ and 396 (25.5%) patients with ER+/PR− were analyzed in our study. The patients with ER+/PR− were more likely to be high histological grade (p < 0.001) and liver metastasis (p = 0.045) compared to patients with ER+/PR+. Despite higher chance of receiving chemotherapy (83.6% vs 77.3%, P = 0.008), patients with ER+/PR− showed worse BCSS (5-year BCSS rate, 34.3% vs 51.3%, P < 0.001) and OS (5-year OS rate, 31.3% vs 46.1%, P < 0.001) compared with ER+/PR+ phenotype. Multivariate survival analysis showed that patients with ER+/PR− still had worse BCSS (hazard ratios [HR]: 1.764, 95% confidence intervals [CI] 1.476–2.109, P < 0.001) and OS (HR: 1.675, 95% CI 1.411–1.975, P < 0.001) than ER+/PR+ phenotype. Furthermore, patients with ER+/PR− showed worse outcomes than ER+/PR+ phenotype in most subgroups, especially in patients with younger age (≤ 60 years), lower histological grade, lymph node involved and distant metastasis. Patients with ER+/PR− had more aggressive biological behaviors and worse outcomes than patients with ER+/PR+ in IBC. Stronger treatments maybe needed for IBC patients with ER+/PR−.

## Introduction

Inflammatory breast cancer (IBC) is a rare subtype and accounts for 2–4% of all breast malignant tumors^1^, but it is characterized by aggressive biological behaviors and accounts for 7% of all breast cancer-related death^2^. Patients with IBC often present rapid progressive pain, erythema, and edema in the involved breast because lymphovascular spaces were embolized by tumor cells. Due to its aggressive behaviors, 85% of the patients already have lymph node involved and 30% of the patients show distant metastasis at the initial diagnosis of IBC^3^. Trimodality treatment including chemotherapy, surgery and radiation therapy has become a widely accepted approach and significantly improved the survival for IBC, but the overall survival (OS) rates remain very low (5-year and 10-year OS rates, 55.4% and 37.3%, respectively)^4^.

As non-IBC, IBC can also be divided into different molecular subtypes according to the status of estrogen receptor (ER), progesterone receptor (PR) and human epidermal growth factor receptor 2 (HER2). Endocrine therapy is recommended for IBC patients with estrogen receptor-positive (ER +) and/or progesterone receptor-positive (PR +) by the National Comprehensive Cancer Network (NCCN) guidelines. Many researches have revealed that the status of PR has great prognostic effect on breast cancer and patients with estrogen receptor-positive/progesterone receptor-positive (ER+/PR +) have better outcomes than patients with estrogen receptor-positive/progesterone receptor-negative (ER+/PR−)^5–7^. However, the role of PR status on IBC has not been illuminated because of the lower incidence of IBC. Actually, IBC is a special subtype and its biologic characteristics are distinct from that of non-IBC. IBC is more likely to be estrogen receptor-negative and HER2-positive compared with non-IBC^8,9^. Besides, some researches have demonstrated that patients with IBC exhibited higher percentage of progesterone receptor-negative status compared with that of patients with non-IBC (55–56.7% versus 32–46.8%)^9,10^. While, it is still unknown whether the absence of PR expression will lead to worse prognosis of IBC or not. Thus, the purpose of this study was to estimate the differences of clinicopathologic features and prognosis between ER+/PR− phenotype and ER+/PR+ phenotype in IBC by analyzing the patients from Surveillance, Epidemiology and End Results (SEER) database.

## Materials and methods

### Patient selection

**Figure 1:**
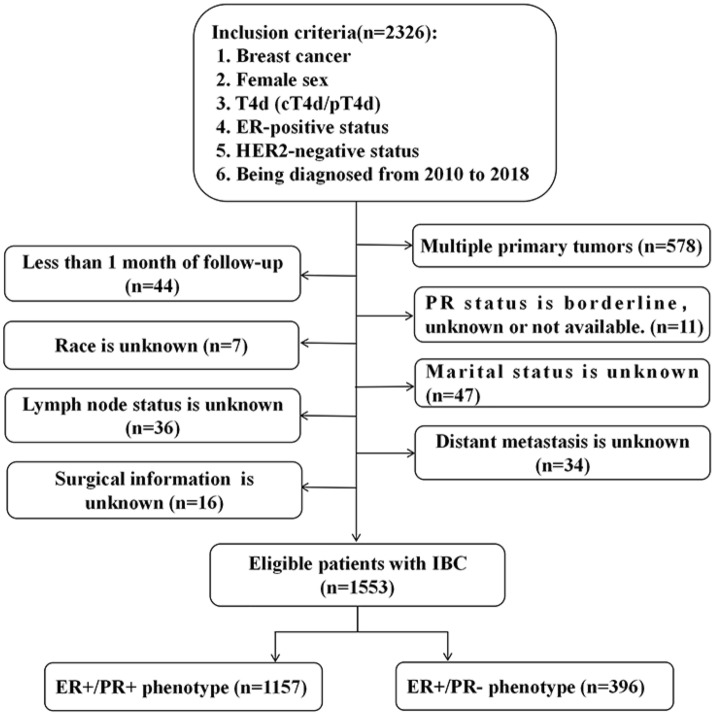
Flowchart for patient selection from the surveillance, epidemiology and end results (SEER) database.

SEER database provides cancer statistics about patients’ demographics, tumor characteristics, methods of treatment and follow-up information, which covers approximately 28% of the United States population. Because the status of HER2 was collected into SEER database since 2010, we used the SEER*Stat version 8.4.0 to identify eligible patients based on the following inclusion criteria: breast cancer, female sex, T4d (cT4d/pT4d), estrogen receptor-positive status, HER2-negtative status and being diagnosed from 2010 to 2018. The excluded criteria were patients with multiple primary tumors, less than 1 month of follow-up, or patients with unknown information about PR status, marital status, lymph node status, distant metastasis and surgery (Fig. 1, flow-chart). Finally, 1553 patients met the criteria and their clinicopathologic data including age, race, marital status, histological grade, lymph node stage, status of PR, sites of distant metastasis, therapeutic regimens and follow-up information were acquired and analyzed. The eligible patients were allocated to two groups (ER+/PR+ phenotype or ER+/PR− phenotype) according the status of PR. After 2010, the status of PR was detected by immunohistochemistry, and negativity was defined as < 1% of malignant cells positively staining for PR^11^.

### Statistical analysis

Pearson’s χ^2^ test was used to estimate the difference of clinicopathologic factors between ER+/PR− phenotype and ER+/PR+ phenotype in IBC. The endpoints were breast cancer-specific survival (BCSS) and overall survival (OS) in our study. BCSS was defined as the interval from the diagnosis of breast cancer to mortality caused by breast cancer or the final follow-up in censored cases. OS was defined as the interval from the diagnosis of breast cancer to mortality from all causes or the final follow-up in censored cases. Survival curves of the patients with ER+/PR− phenotype or ER+/PR+ phenotype were constructed by the Kaplan–Meier method, and the log-rank test was applied to determine the effect of PR status on BCSS and OS. A Cox proportional hazards model was used for the multivariate analysis and to estimate hazard ratios with 95% confidence intervals (CIs). STATA software (Version 13; Stata Corporation) was applied for all statistical analyses. The forest plot was generated by Microsoft Office Excel (Version 2021; Microsoft Corporation). All tests were two sided and p-value < 0.05 were considered statistically significant.

### Ethical approval

This study used previously collected de-identified data, and the need for informed consent had been waived due to the retrospective nature of the study, and was deemed exempt from review by the Ethics Committee of the Affiliated Hospital of North Sichuan Medical College.

## Results

### Clinicopathologic features

A total of 1553 patients with ER+ and HER2-negative IBC met the criteria and were analyzed in our study. Among them, 1157 (74.5%) patients were ER+/PR+ phenotype and 396 (25.5%) patients were ER+/PR− phenotype. As shown in Table 1, the patients with ER+/PR− phenotype were more likely to be high histological grade (III-IV) compared with ER+/PR+ phenotype (59.8% and 44%, P < 0.001). More bone metastasis happened to patients with ER+/PR+ than patients with ER+/PR− phenotype (29% and 24.5%, P = 0.082), but no statistical difference was reached. While, more liver metastasis happened to patients with ER+/PR− than patients with ER+/PR+ phenotype (11.4% and 8%, P = 0.045). More patients with ER+/PR− phenotype received chemotherapy than patients with ER+/PR+ phenotype (83.6% and 77.3%, P < 0.001). There were no significant differences between ER+/PR− phenotype and ER+/PR+ phenotype in terms of age, race, marital status, lymph node stage, TNM stage, lung metastasis, brain metastasis, surgery and radiation.

**Table 1:** The clinicopathological features of patients with ER-positive and HER2-negative IBC.

Variables	Total number		ER+/PR+		ER+/PR−		P-value
			N	%	N	%	
All patients	1553		1157	74.5	396	25.5	
Age (years)							
≤ 60	885	651	56.3		234	59.1	0.321
> 60	668	506	43.7		162	40.9	
Race							
White	1178	886	76.6		292	73.7	0.098
Black	263	183	15.8		80	20.2	
Others	112	88	7.6		24	6.1	
Marital status							
Married	714	531	45.9		183	46.2	0.913
Unmarried	839	626	54.1		213	53.8	
Grade							
I–II	607	499	43.1		108	27.3	< 0.001
III–IV^#^	746	509	44.0		237	59.8	
Unknown	200	149	12.9		51	12.9	
Lymph node stage							
N_0_	189	138	11.9		51	12.9	0.343
N_1_	708	543	46.9		165	41.6	
N_2_	320	233	20.2		87	22.0	
N_3_	336	243	21.0		93	23.5	
TNM stage							
III	942	700	60.5		242	61.1	0.83
IV	611	457	39.5		154	38.9	
Bone metastasis							
No	1120	821	71.0		299	75.5	0.082
Yes	433	336	29.0		97	24.5	
Liver metastasis							
No	1415	1064	92.0		351	88.6	0.045
Yes	138	93	8.0		45	11.4	
Lung metastasis							
No	1356	1001	86.5		355	89.6	0.106
Yes	197	156	13.5		41	10.4	
Brain metastasis							
No	1515	1132	97.8		383	96.7	0.212
Yes	38	25	2.2		13	3.3	
Surgery							
BCS	43	32	2.8		11	2.8	0.997
Mastectomy	913	682	58.9		231	58.3	
None	597	443	38.3		154	38.9	
Radiation							
Yes	819	616	53.2		203	51.3	0.496
No	734	541	46.8		193	48.7	
Chemotherapy							
Yes	1225	894	77.3		331	83.6	0.008
No	328	263	22.7		65	16.4	

^#^Grade IV refers to breast cancer with undifferentiated or anaplastic histological features. *ER* Estrogen receptor, *PR* Progesterone receptor, *IBC* Inflammatory breast cancer, *BCS* Breast-conserving surgery.

### Univariate survival analysis

**Figure 2:**
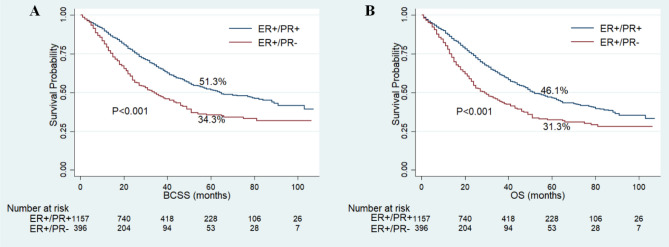
Kaplan–Meier curves of breast cancer-specific survival (**A**) and overall survival (**B**) based on hormone receptor status for patients with ER-positive and HER2-negative inflammatory breast cancer.

**Figure 3:**
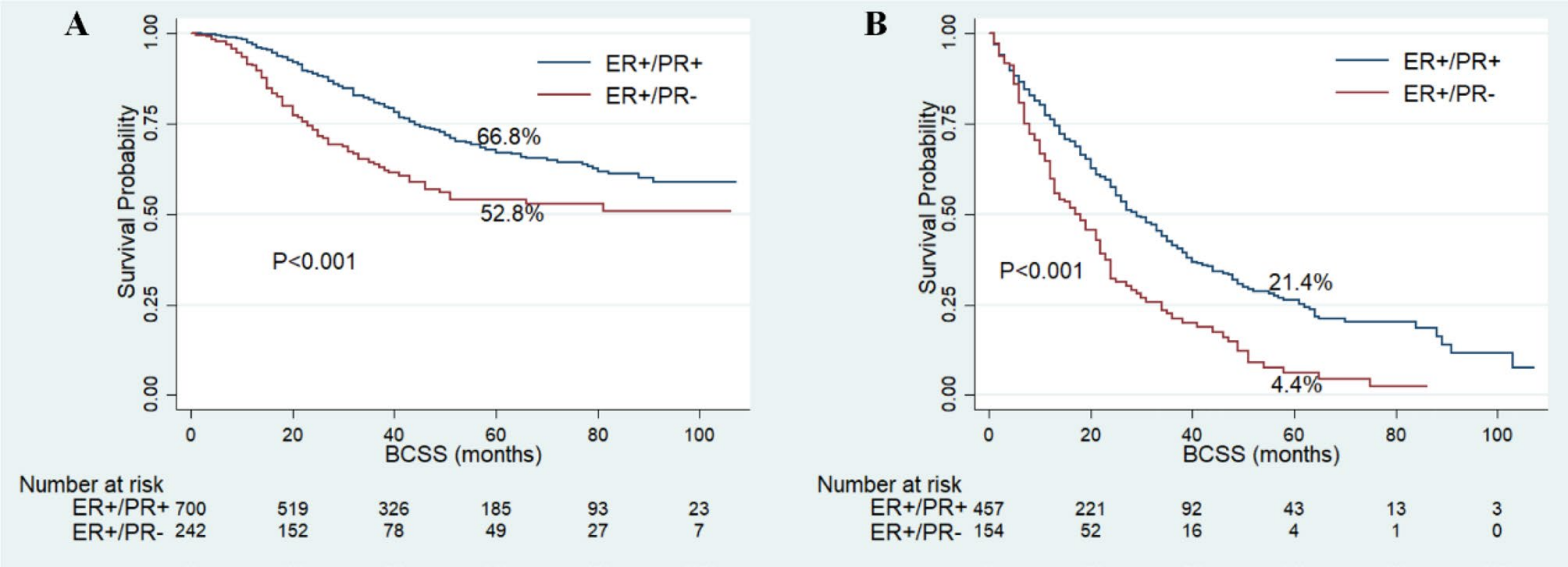
Kaplan–Meier curves of breast cancer-specific survival based on hormone receptor status for patients with ER-positive and HER2-negative inflammatory breast cancer in stage III (**A**) and stage IV (**B**).

**Figure 4:**
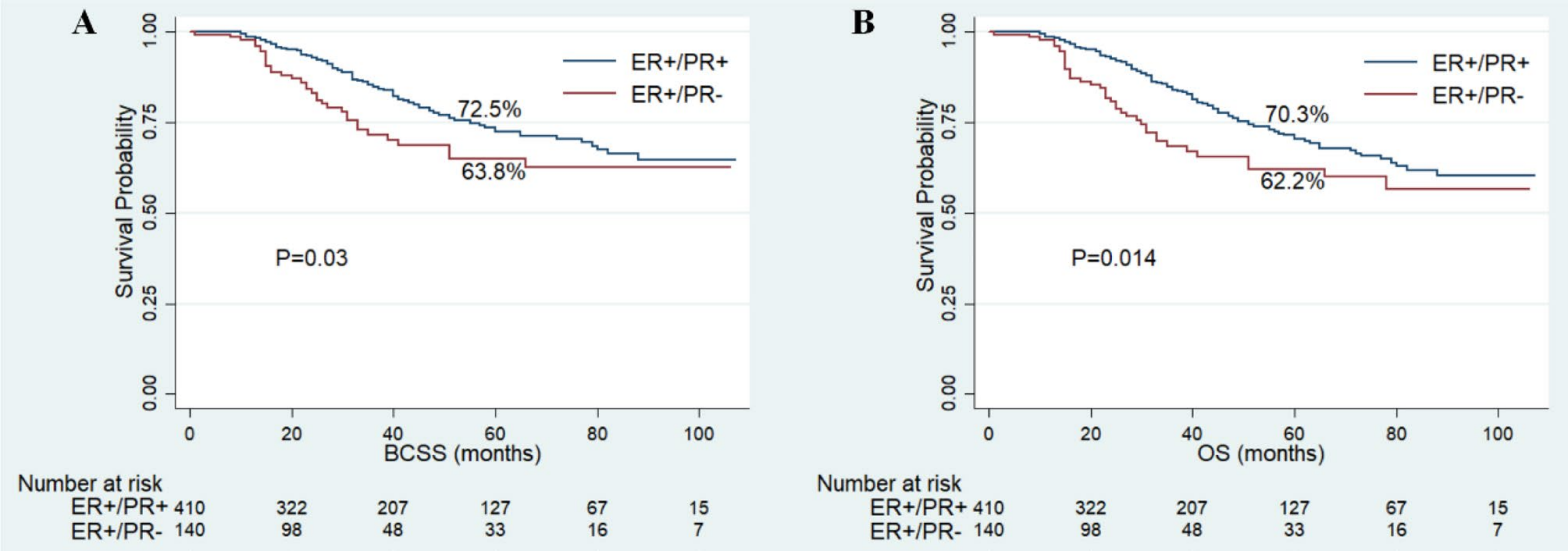
Kaplan–Meier curves of breast cancer-specific survival (**A**) and overall survival (**B**) based on hormone receptor status for stage III inflammatory breast cancer patients with ER-positive and HER2-negative after standard trimodality treatment.

The follow-up time of this cohort ranged from 1 to 107 months, with a median of 25 months. Finally, 724 patients had died and 631 cases of the death were attributed to IBC. The 5-year BCSS and OS rates were 47.3% and 42.5%, respectively. Among the whole cohort, the patients with ER+/PR− phenotype showed worse BCSS (5-year BCSS rate, 34.3% vs 51.3%, P < 0.001) and OS (5-year OS rate, 31.3% vs 46.1%, P < 0.001) compared with patients of ER+/PR+ phenotype (Fig. 2). As shown in Fig. 3, the patients with ER+/PR− phenotype had worse BCSS than patients with ER+/PR+ phenotype in stage III (5-year BCSS rate, 52.8% vs 66.8%, P < 0.001) and stage IV (5-year BCSS rate, 4.4% vs 21.4%, P < 0.001) IBC. After the standard trimodality treatment (chemotherapy, surgery and radiation therapy), the patients with ER+/PR− phenotype still exhibited worse BCSS (5-year BCSS rate, 63.8% vs 72.5%, P = 0.03) and OS (5-year OS rate, 62.2% vs 70.3%, P = 0.014) than patients with ER+/PR+ phenotype in stage III IBC (Fig. 4).

### Multivariate survival analysis

Multivariate survival analysis showed that the patients with ER+/PR− phenotype still had worse BCSS (HR: 1.764, 95% CI 1.476–2.109, P < 0.001) and OS (HR: 1.675, 95% CI 1.411–1.975, P < 0.001) than patients with ER+/PR+ phenotype (Table 2). Then, older age (> 60 years), unmarried status, black race, higher histological grade (III–IV), higher lymph node stage (N3) and distant metastasis were independent risk factors for BCSS and OS in patients with ER+ and HER2-ngeative IBC. Breast surgery, radiation and chemotherapy could significantly improve the survival for IBC.

**Table 2:** Multivariable Cox regression for BCSS and OS among patients with ER-positive and HER2-negative IBC.

Variables	Total number	BCSS			OS		
		HR	95% CI	P-value	HR	95% CI	P-value
Age (years)							
≤ 60	885	Ref			Ref		
> 60	668	1.325	1.118–1.570	0.001	1.405	1.199–1.646	< 0.001
Race							
White	1178	Ref			Ref		
Black	263	1.368	1.120–1.670	0.002	1.365	1.132–1.645	< 0.001
Others	112	0.76	0.543–1.063	0.109	0.718	0.520–0.992	0.044
Marital status							
Married	714	Ref			Ref		
Unmarried	839	1.212	1.028–1.430	0.022	1.274	1.092–1.488	0.002
Grade							
I–II	607	Ref			Ref		
III–IV	746	1.389	1.161–1.663	< 0.001	1.419	1.200–1.678	< 0.001
Unknown	200	1.449	1.135–1.849	0.003	1.445	1.151–1.815	0.002
HoR status							
ER+/PR+	1157	Ref			Ref		
ER+/PR−	396	1.764	1.476–2.109	< 0.001	1.675	1.411–1.975	< 0.001
Lymph node stage							
N_0_	189	Ref			Ref		
N_1_	708	1.173	0.899–1.530	0.239	1.245	0.973–1.593	0.081
N_2_	320	1.054	0.781–1.423	0.729	1.029	0.777–1.363	0.843
N_3_	336	1.516	1.135–2.024	0.005	1.544	1.178–2.024	0.002
TNM Stage							
III	942	Ref			Ref		
IV	611	1.983	1.524–2.579	< 0.001	1.705	1.328–2.189	< 0.001
Bone metastasis							
No	1120	Ref			Ref		
Yes	433	1.504	1.185–1.907	0.001	1.554	1.235–1.955	0.001
Liver metastasis							
No	1415	Ref			Ref		
Yes	138	2.027	1.594–2.578	< 0.001	2.004	1.591–2.526	< 0.001
Lung metastasis							
No	1356	Ref			Ref		
Yes	197	1.24	0.992–1.549	0.059	1.178	0.950–1.461	0.135
Brain metastasis							
No	1515	Ref			Ref		
Yes	38	1.188	0.775–1.821	0.43	1.319	0.886–1.964	0.172
Surgery							
Mastectomy	913	Ref			Ref		
BCS	43	1.22	0.733–2.031	0.975	1.139	0.705–1.842	0.595
None	597	1.282	1.037–1.585	0.021	1.254	1.029–1.528	0.025
Radiation							
No	734	Ref			Ref		
Yes	819	0.792	0.662–0.949	0.011	0.749	0.633–0.886	0.001
Chemotherapy							
No	328	Ref			Ref		
Yes	1225	0.624	0.513–0.759	< 0.001	0.583	0.486–0.698	< 0.001

*HoR* hormone receptor, *ER* estrogen receptor, *PR* progesterone receptor, *IBC* inflammatory breast cancer, *BCS* breast-conserving surgery, *BCSS* breast cancer-specific survival, *OS* overall survival, *Ref* reference, *HR* hazard ratios, *CI* confidence intervals.

### Subgroup survival comparation for ER+/PR+ and ER+/PR− phenotypes

**Figure 5:**
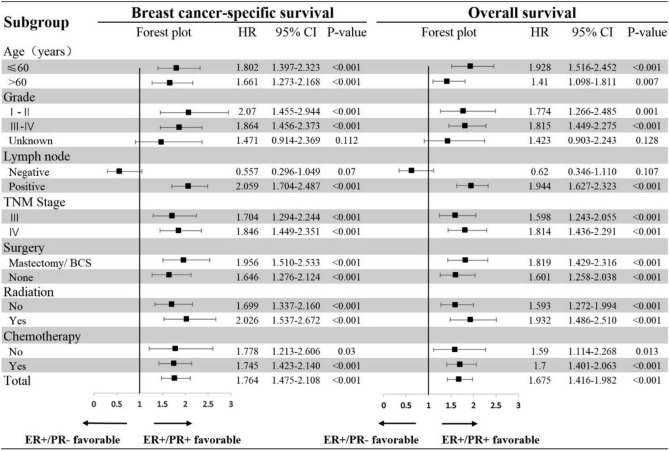
Subgroup survival analysis of the multivariate Cox regression models. It shows the difference in breast cancer-specific survival and overall survival between ER+/PR− phenotype and ER+/PR+ phenotype in inflammatory breast cancer.

When subgroup analysis was performed by multivariate Cox regression models (Fig. 5), the patients with ER+/PR− phenotype also showed worse outcomes than patients with ER+/PR+ phenotype in most subgroups. Especially in patients with younger age (≤ 60 years) (BCSS, HR: 1.802, 95% CI 1.397–2.323, P < 0.001; OS, HR: 1.928, 95% CI 1.516–2.452, P < 0.001), lower histological grade (BCSS, HR: 2.07, 95% CI 1.455–2.944, P < 0.001), lymph node involved (BCSS, HR: 2.059, 95% CI 1.704–2.487, P < 0.001; OS, HR: 1.944, 95% CI 1.627–2.323, P < 0.001) and distant metastasis (BCSS, HR: 1.846, 95% CI 1.449–2.351, P < 0.001; OS, HR: 1.814, 95% CI 1.436–2.291, P < 0.001), significant worse prognoses were seen in ER+/PR− phenotype compared with ER+/PR+ phenotype.

## Discussion

Progesterone receptor (PR) is a downstream gene target of estrogen receptor (ER) and the loss of PR often indicates a poor prognosis in breast cancer^7,12,13^. Previous studies have shown that ER+/PR− phenotype accounts for 10.5–15% of ER-positive breast cancer^6,7,12,14^. However, it’s worth noting that 25.5% of the patients were ER+/PR− IBC in our study and this proportion is much higher than that of the whole breast cancer population, which may partly explain the worse outcome of IBC. Keeping with previous study^3^, 87.8% of the patients already had lymph node involved and 39.3% of the patients were distant metastasis at the initial diagnosis of breast cancer, which demonstrated the aggressive biological behavior of IBC. On accounting of higher proportion of patients with ER+/PR− phenotype and more aggressive biological characteristics, it is very necessary to figure out the effect of PR status on IBC.

As previous studies^6,7,15^, the loss of PR also predicted unfavorable biological characteristics in IBC. In our study, the patients with ER+/PR− phenotype were prone to be poor histological grade (III-IV), which often predicts worse survival^16,17^. In addition, the tendency of distant metastasis for ER+/PR− phenotype differed from ER+/PR+ phenotype. Consistent with our previous study^15^, more liver metastasis happened to patients with ER+/PR− phenotype than patients with ER+/PR+ phenotype in IBC, which indicates greater propensity of visceral metastasis for ER+/PR− phenotype. However, more bone metastasis occurred to patients with ER+/PR+ phenotype than patients with ER+/PR− phenotype, which demonstrated a pattern of bone metastatic spread typically attributed to hormone receptor-positive breast cancer^18^.

Although the prognosis of breast cancer has been greatly improved with the advent of various systemic treatments, the survival of IBC was still far from satisfaction. Consistent with previous studies^19,20^, the survival for IBC was very poor in our research (5-year BCSS and OS rates, 47.3% and 42.5%, respectively). Given the poor prognosis for IBC, previous researches tried to find the risk factors and demonstrated many independent predicted factors, such as race, lymph node ratio, AJCC stage, histological grade, ER status, PR status, HER2 status, surgery status, and radiotherapy status^16,19^. Although those researches have demonstrated that PR-negative status contributes to worse outcome for IBC, the difference of prognosis between ER+/PR− phenotype and ER+/PR+ phenotype is still unknown and needed to be further verified because worse outcome of PR-negative cohort mainly resulted from patients with triple-negative IBC in their study. Therefore, we excluded triple-negative IBC and compared the prognosis between ER+/PR− phenotype and ER+/PR+ phenotype in IBC by those two cohorts. As shown in our study, the patients with ER+/PR− phenotype exhibited significant worse survival than patients with ER+/PR+ phenotype, especially in the patients with younger age, lower histological grade, lymph node involved and distant metastasis. Due to the worse clinicopathologic features and prognosis for patients with ER+/PR− phenotype, the loss of PR has aroused wide attention from scholars. The main researches focused on genomics changes, such as PR promoter hypermethylation or loss of heterozygosity at the PR gene locus^21,22^. A recent study illuminated that almost 20% of the patients with ER+/PR− and HER2-negative were non-luminal-like and didn’t benefit from sufficient endocrine therapy^12^, which partly explains the worse outcome for IBC with ER+/PR− phenotype. As mentioned above, higher percentage of patients presented PR loss in IBC and worse survival were seen in those patients. Therefore, the patients with ER+/PR− phenotype belonging to non-luminal-like IBC should also be identified and more effective treatments should be performed on them, such as cyclin-dependent kinase 4/6 inhibitors. In addition to PR status, older age and black race were also prognostic risk factors for patients with ER+ and HER2-negative IBC. Poor histological grade and visceral metastasis were recognized as poor prognostic factors for IBC^16,19^, which was also demonstrated by our study. As shown above, the patients with ER+/PR− IBC presented poor histological grade and more visceral metastasis, which may also explain the worse outcomes for patients with ER+/PR− phenotype in our study. Chemotherapy, surgery and radiation are the indispensable approaches of trimodality treatment^23^, and all of these could significantly improve the survival for IBC in our study.

The limitations of this study must be clarified. First, some bias can’t be avoided for the nature of retrospective study. Thus, multivariable Cox proportional hazards model and subgroup analysis were performed to adjust for confounding effects as much as possible. Second, the information about endocrine therapy can’t be acquired from the database, which impeded the further analysis about the effectiveness of endocrine therapy. Nevertheless, most of the patients should have received enough endocrine therapy for the widespread of standard treatment in the United States. Third, because the SEER database didn’t collect the follow-up information about local recurrence and distant metastasis, we can’t analyze the recurrence-free survival and distant disease-free survival. However, our study is the first one that used the relatively large cohort to estimate the influence of PR status on patients with IBC. It illuminated the significant discordance of clinicopathological features and prognosis between ER+/PR− phenotype and ER+/PR+ phenotype in IBC, which indicated the necessity that stronger treatments should be applied to patients with ER+/PR− IBC.

## Conclusions

More than 25% of the patients presented loss of PR expression among the ER-positive and HER2-negative IBC. Poor prognostic factors were prone to occur in IBC patients with ER+/PR− phenotype than ER+/PR+ phenotype, such as higher histological grade (III–IV) and liver metastasis. More effective treatments should be applied to IBC patients with ER+/PR− phenotype because significant worse outcomes were seen in those patients compared with ER+/PR+ phenotype.

## Data Availability

The datasets presented in this study can be found in online repository: the Surveillance, Epidemiology, and End Results (SEER) database (https://seer.cancer.gov).

## Abbreviations

HoR: Hormone receptor
PR: Progesterone receptor
ER: Estrogen receptor
HER2: Human epidermal growth factor receptor 2
IBC: Inflammatory breast cancer
BCSS: Breast cancer-specific survival
OS: Overall survival
SEER: Surveillance, epidemiology and end results
CIs: Confidence intervals
BCS: Breast-conserving surgery
Ref: Reference
HR: Hazard ratios
